# Age-related changes in behavior in C57BL/6J mice from young adulthood to middle age

**DOI:** 10.1186/s13041-016-0191-9

**Published:** 2016-01-28

**Authors:** Hirotaka Shoji, Keizo Takao, Satoko Hattori, Tsuyoshi Miyakawa

**Affiliations:** Division of Systems Medical Science, Institute for Comprehensive Medical Science, Fujita Health University, 1-98 Dengakugakubo, Kutsukake-cho, Toyoake, Aichi 470-1192 Japan; Japan Science and Technology Agency, Core Research for Evolutional Science and Technology, 4-1-8 Honcho, Kawaguchi, Saitama 332-0012 Japan; Section of Behavior Patterns, Center for Genetic Analysis of Behavior, National Institute for Physiological Sciences, 5-1 Higashiyama, Myodaiji-cho, Okazaki, Aichi 444-8787 Japan

**Keywords:** Aging, Behavior, Behavioral test battery, C57BL/6J, Mice

## Abstract

**Background:**

Aging is considered to be associated with progressive changes in the brain and its associated sensory, motor, and cognitive functions. A large number of studies comparing young and aged animals have reported differences in various behaviors between age-cohorts, indicating behavioral dysfunctions related to aging. However, relatively little is known about behavioral changes from young adulthood to middle age, and the effect of age on behavior during the early stages of life remains to be understood. In order to investigate age-related changes in the behaviors of mice from young adulthood to middle age, we performed a large-scale analysis of the behavioral data obtained from our behavioral test battery involving 1739 C57BL/6J wild-type mice at 2–12 months of age.

**Results:**

Significant behavioral differences between age groups (2–3-, 4–5-, 6–7-, and 8–12-month-old groups) were found in all the behavioral tests, including the light/dark transition, open field, elevated plus maze, rotarod, social interaction, prepulse inhibition, Porsolt forced swim, tail suspension, Barnes maze, and fear conditioning tests, except for the hot plate test. Compared with the 2–3-month-old group, the 4–5- and 6–7-month-old groups exhibited decreased locomotor activity to novel environments, motor function, acoustic startle response, social behavior, and depression-related behavior, increased prepulse inhibition, and deficits in spatial and cued fear memory. For most behaviors, the 8–12-month-old group showed similar but more pronounced changes in most of these behaviors compared with the younger age groups. Older groups exhibited increased anxiety-like behavior in the light/dark transition test whereas those groups showed seemingly decreased anxiety-like behavior measured by the elevated plus maze test.

**Conclusions:**

The large-scale analysis of behavioral data from our battery of behavioral tests indicated age-related changes in a wide range of behaviors from young adulthood to middle age in C57BL/6J mice, though these results might have been influenced by possible confounding factors such as the time of day at testing and prior test experience. Our results also indicate that relatively narrow age differences can produce significant behavioral differences during adulthood in mice. These findings provide an insight into our understanding of the neurobiological processes underlying brain function and behavior that are subject to age-related changes in early to middle life. The findings also indicate that age is one of the critical factors to be carefully considered when designing behavioral tests and interpreting behavioral differences that might be induced by experimental manipulations.

**Electronic supplementary material:**

The online version of this article (doi:10.1186/s13041-016-0191-9) contains supplementary material, which is available to authorized users.

## Background

Aging is a complex process associated with structural and physiological changes in the brain that can account for age-related behavioral changes and increased incidence of neuropsychiatric disorders. Rodents have been extensively used as models for human aging and disease because of their widespread availability and short life span. A number of rodent studies have examined the impact of age on brain and behavior, contributing to our understanding of the brain mechanisms underlying behavioral changes associated with normal and pathological aging [1–3]. Many such age-related behavioral differences have been reported from comparisons between young adult (2–6 months of age) and aged (18 months of age and over) animals through behavioral tests (e.g., [4–13]). In the past two decades, advances in gene targeting technology have enabled us to generate targeted gene mutations in mice, which has increased interest in the use of mutant mouse models to elucidate the relationship between the aging brain and behavior (e.g., [14, 15]). Despite these extensive studies comparing young and aged animals and the increasing interest in mutant models, there is still relatively little information regarding age-related changes in behavior from young adulthood to middle age (approximately 2–12 months of age) in the background strains of mice used to create these mutant mice.

Behavioral test batteries have been used to assess a wide variety of behavioral traits, including locomotor activity, sensory and motor functions, anxiety-like behavior, and learning and memory, in a cohort of inbred and mutant mouse strains [16–21]. Most of the behavioral traits were sensitive to genetic, environmental, and experimental factors, e.g., genetic background, laboratory conditions, and prior test experience [22–27]. Thus, behavioral experiments are generally designed to minimize the potential effects of various confounding factors, and the use of a battery of standardized behavioral tests is needed to ensure more accurate interpretations of behavioral phenotypes. In our laboratory, we have examined over 170 strains of genetically engineered mice using a comprehensive behavioral test battery according to our standardized protocol [21]. The large amount of behavioral data from various cohorts of mice of different ages may be useful for understanding the effects of age on mouse behavior.

The present study was conducted in order to examine the effects of age on behavior from young adulthood to middle age, and to identify behaviors that are affected by age at the early stages of life. We performed a large-scale analysis of behavioral data obtained from mice of different ages using our behavioral test battery. Our test battery included general health and neurological screening, light/dark transition, open field, elevated plus maze, hot plate, social interaction, rotarod, startle response/prepulse inhibition, Porsolt forced swim, Barnes maze, fear conditioning, and tail suspension tests. These behavioral tests had been performed in a nearly uniform order following our standardized protocols. We used the behavioral data from up to 1739 C57BL/6J wild-type mice, which is a widely used inbred strain that often serves as a background strain for mutant mice, at 2–12 months of age. The detailed characterization of age-related changes in behavior of C57BL/6J mice will provide researchers with useful information for designing behavioral experiments, interpreting mouse phenotypes, and understanding the neurobiological basis of age-related behavioral changes. Our large-scale analysis showed significant behavioral differences between age groups in almost all the tests, demonstrating the effects of age on various behavioral domains in C57BL/6J mice, from young adulthood to middle age.

## Results

**Fig. 1:**
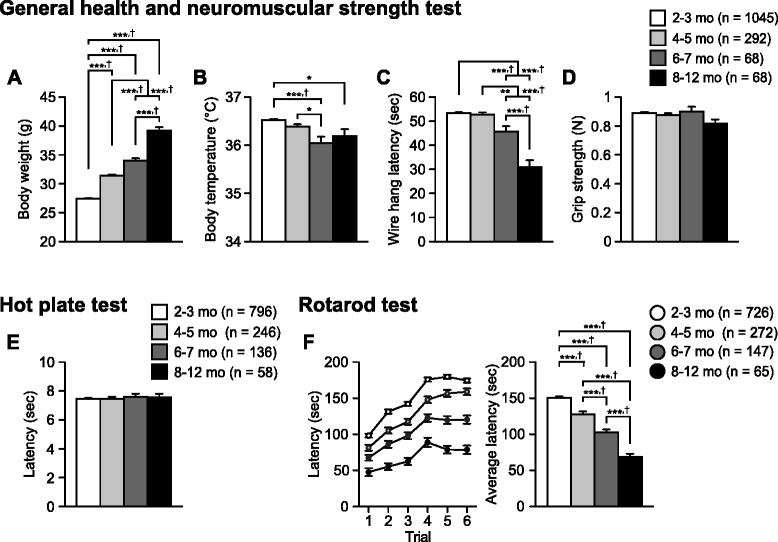
Increased body weight, decreased muscular strength, normal pain sensitivity, and motor dysfunction in older C57BL/6J mice. **a** Body weight (g), **b** body temperature (°C), **c** wire hang latency (s), **d** grip strength (Newton, N), **e** latency to paw lick or foot shake (s) in the hot plate test, and **f** latency to fall off a rotating rod (s) in the rotarod test. Values are means ± SEM. *p < 0.05, **p < 0.01, and ***p < 0.001 after Bonferroni correction performed when ANOVAs reached nominal significance. †*p* < 0.05 after Bonferroni correction performed when ANOVAs reached study-wide significance

**Fig. 2:**
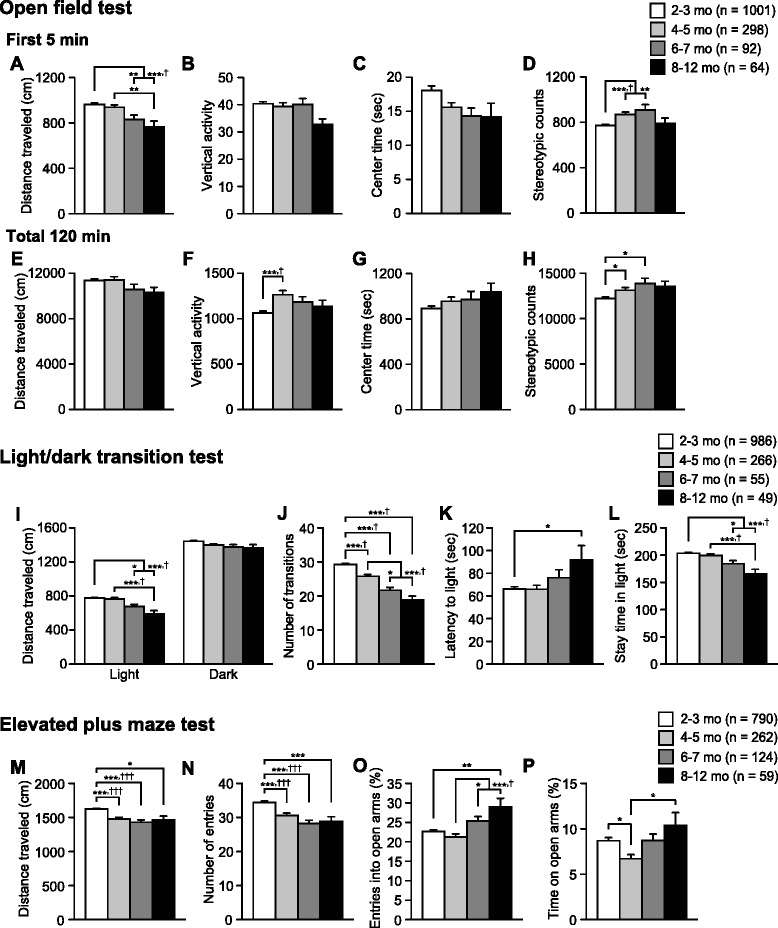
Decreased locomotor activity and altered anxiety-like behavior in older C57BL/6J mice. **a**-**h** Open field test: **a**, **e** distance traveled (cm), **b**, **f** vertical activity, **c**, **g** center time (s), and **d**, **h** stereotypic counts during the first 5-min and the entire 120-min period of the test. **i**-**l** Light/dark transition test: **i** distance traveled (cm) in the light and dark chambers, **j** number of transitions, **k** latency to enter the light chamber (s), and **l** time spent in the light chamber (s). **m**-**p** Elevated plus maze test: **m** Distance traveled (cm), **n** number of arm entries, **o** entries into open arms (%), and **p** time spent in open arms (%). Values are means ± SEM. *p < 0.05, **p < 0.01, and ***p < 0.001 after Bonferroni correction performed when ANOVAs reached nominal significance. †*p* < 0.05 after Bonferroni correction performed when ANOVAs reached study-wide significance

**Fig. 3:**
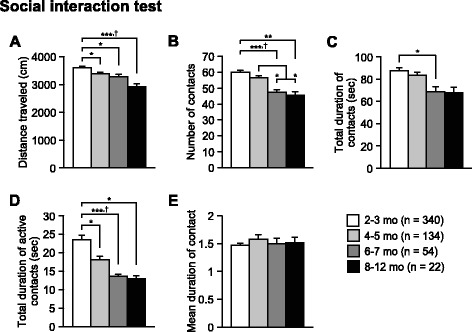
Decreased social contacts in older C57BL/6J mice. **a** Distance traveled (cm), **b** number of contacts, **c** total duration of contacts (s), **d** total duration of active contacts (s), and **e** mean duration of contact (s). Values are means ± SEM. *p < 0.05, **p < 0.01, and ***p < 0.001 after Bonferroni correction performed when ANOVAs reached nominal significance. †*p* < 0.05 after Bonferroni correction performed when ANOVAs reached study-wide significance

**Fig. 4:**
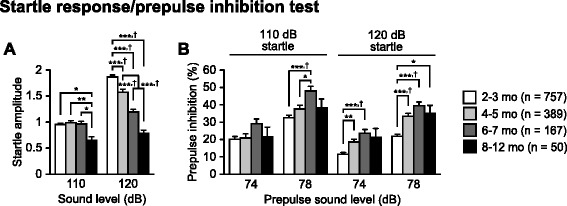
Age-related changes in acoustic startle response and prepulse inhibition in C57BL/6J mice. **a** Acoustic startle response to sound stimuli (110 and 120 dB white noise) and **b** prepulse inhibition (%) of the startle response with 74 and 78 dB prepulse stimuli. Values are means ± SEM. *p < 0.05, **p < 0.01, and ***p < 0.001 after Bonferroni correction performed when ANOVAs reached nominal significance. †*p* < 0.05 after Bonferroni correction performed when ANOVAs reached study-wide significance

**Fig. 5:**
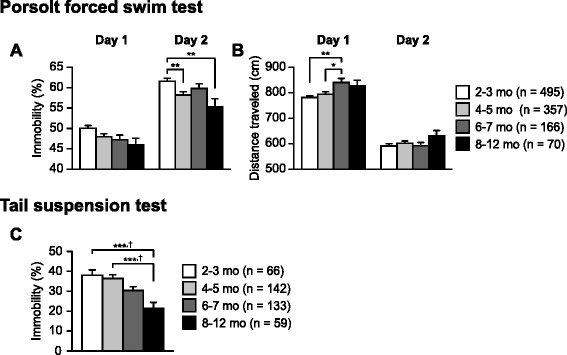
Decreased immobility in older C57BL/6J mice. **a** Immobility time (%) and **b** distance traveled (cm) in the Porsolt forced swim test. **c** Immobility time (%) in the tail suspension test. Values are means ± SEM. *p < 0.05, **p < 0.01, and ***p < 0.001 after Bonferroni correction performed when ANOVAs reached nominal significance. †*p* < 0.05 after Bonferroni correction performed when ANOVAs reached study-wide significance

**Fig. 6:**
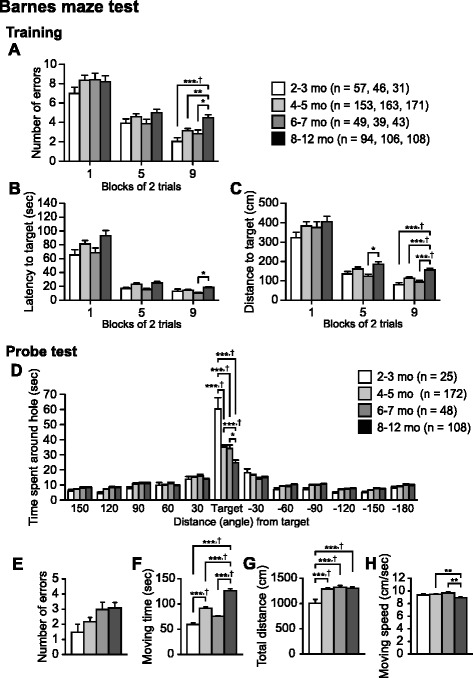
Impaired spatial learning and memory in older C57BL/6J mice. **a** Number of errors to reach the target hole, **b** latency to reach the target hole, and **c** distance traveled to reach the target hole during the training session of the Barnes maze test. **d** Time spent around the target hole in the probe test. **e** Number of errors to reach the target hole, **f **moving time (s), **g** total distance traveled (cm), and **h** moving speed (cm/s) in the probe test. Values are means ± SEM. *p < 0.05, **p < 0.01, ***p < 0.001 after Bonferroni correction performed when ANOVAs reached nominal significance. †*p* < 0.05 after Bonferroni correction performed when ANOVAs reached study-wide significance

**Fig. 7:**
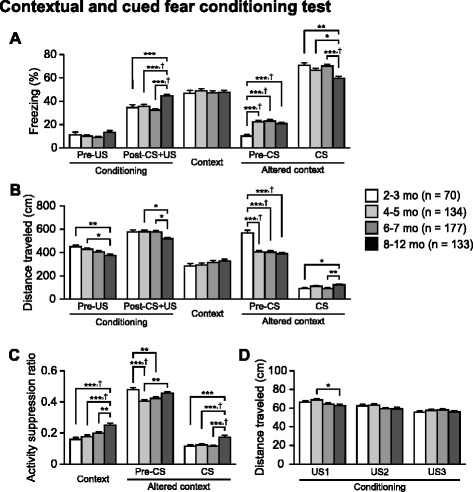
Reduced contextual and cued fear memory in older C57BL/6J mice. **a** Freezing (%) and **b** distance traveled (cm) in the conditioning, context test, and cued test. **c** Activity suppression ratio in the context and cued tests. **d** Distance traveled (cm) was measured during each footshock. Values are means ± SEM. *p < 0.05, **p < 0.01, and ***p < 0.001 after Bonferroni correction performed when ANOVAs reached nominal significance. †*p* < 0.05 after Bonferroni correction performed when ANOVAs reached study-wide significance

We divided the behavioral data from up to 1739 wild-type C57BL/6J mice into the following four age groups for each behavioral test: 2–3, 4–5, 6–7, and 8–12 months of age. The data from each behavioral test were statistically compared between the age groups using analysis of variance (Additional file 1: Table S1). We defined “study-wide significance” as the statistical significance that survived Bonferroni correction for 69 behavioral measures used in the analysis (*p* < 0.05/69 = 0.000724). “Nominal significance” was defined as the one that achieved a statistical significance in an index (*p* < 0.05) but did not survive the correction. The *post-hoc* multiple comparisons were further performed using Bonferroni correction (for the study wide significance, *p* < 0.000724/6 = 0.00012; for the nominal significance, *p* < 0.05/6 = 0.008333). Unless otherwise noted, the results for nominal significance were described in the following sections (see Figs. 1, 2, 3, 4, 5, 6 and 7, for the results of *post-hoc* analyses with Bonferroni correction after the study-wide significance was obtained).

### Increased body weight, reduced neuromuscular strength, normal pain sensitivity, and motor dysfunction in older C57BL/6J mice

We examined age-related changes in physical characteristics and neuromuscular strength. One-way analysis of variances (ANOVAs) revealed that there were significant effects of Age on body weight (Fig. 1a; F_3,1469_ = 456.49, *p* < 0.0001), body temperature (Fig. 1b; F_3,1469_ = 9.86, *p* < 0.0001), and wire hang latency (Fig. 1c; F_3,1469_ = 51.47, *p* < 0.0001), all of which reached study-wide significance. *Post-hoc* tests showed that subjects in the older age groups were significantly heavier than those in the younger age groups (8–12-month-old mice [8–12 mo] > 6–7-month-old mice [6–7 mo] > 4–5-month-old mice [4–5 mo] > 2–3-month-old mice [2–3 mo], all comparisons *p* < 0.0001). The body temperatures of older age groups were significantly lower than those of the younger age groups (8–12 and 6–7 mo < 2–3 mo, *p* = 0.0024 and *p* < 0.0001, respectively; 6–7 mo < 4–5 mo, *p* = 0.0037). In the wire hang test, subjects in the older age groups showed a shorter latency to fall off the wire than those in the younger age groups (8–12 mo < 6–7 mo, *p* < 0.0001; 8–12 and 6–7 mo < 4–5 mo, *p* < 0.0001 and *p* = 0.0004, respectively; 8–12 and 6–7 mo < 2–3 mo, *p* < 0.0001 and *p* < 0.0001, respectively). Regarding the grip strength, although there was no significant effect of Age (Fig. 1d; F_3,1469_ = 2.44, *p* = 0.0631), subjects in the oldest age group exhibited lower grip strengths than those in younger age groups (8–12 mo < 6–7, 4–5, and 2–3 mo, *p* = 0.031, *p* = 0.0561, and *p* = 0.011, respectively). These results indicate that muscular strength decreases with age.

The hot plate test is widely used to assess pain sensitivity to a thermal stimulus. In this test, there was no significant effect of Age on paw responses to a thermal stimulus (Fig. 1e; F_3,1232_ = 0.25, *p* = 0.8644). This result indicates no age-related changes in pain sensitivity.

The rotarod test, in which mice are required to walk on an accelerating rotating rod across six trials, is used to evaluate motor function. Average latency to fall off the rod was calculated from the six trials. ANOVA revealed a significant effect of Age on the average latency (Fig. 1f; F_3,1206_ = 62.54, *p* < 0.0001), which reached study-wide significance. Older mice showed a significantly shorter latency to fall off the rod compared with younger mice (8–12 mo < 6–7 mo < 4–5 mo < 2–3 mo, all comparisons *p* < 0.0001). Previous studies have indicated that rotarod performance is negatively correlated with body weight [28, 29]. We performed an analysis of covariance (ANCOVA) with body weight measured at the beginning of the test battery as a covariate. This analysis revealed a significant Age effect and a significant Age × Body weight interaction in the average rotarod latency (Age effect, F_3,1205_ = 15.059, *p* < 0.0001; Body weight effect, F_1,1202_ = 31.48, *p* < 0.0001; Age × Body weight, F_3,1202_ = 3.899, *p* = 0.0087), indicating that the linear association between rotarod performance and body weight varies with ages. In older age groups, there were negative correlations between body weight and average rotarod latency (Additional file 2: Figure S1A-E; all age groups, *r* = −0.352, *p* < 0.0001; 2–3 mo, *r* = −0.039, *p* = 0.2997; 4–5 mo, *r* = −0.289, *p* < 0.0001; 6–7 mo, *r* = −0.153, *p* = 0.0645; 8–12 mo, *r* = −0.68, *p* < 0.0001). To control the effect of body weight, we used the data from mice with body weights ranging from 27.5 to 32.5 g and compared the rotarod latency among age groups. ANOCOVA showed that there was a significant effect of Age on the rotarod latency (Additional file 2: Figure S1F, G; Age effect, F_3,522_ = 12.37, *p* < 0.0001; Body weight effect, F_1,522_ = 0.05, *p* = 0.8182). These data suggest that aging is associated with decreased motor function.

### Decreased locomotor activity and altered anxiety-like behaviors in older C57BL/6J mice

The open field test is widely used to measure locomotor activity and anxiety-like behavior [30]. In the first 5-min period of the test, there were significant effects of Age on the distance traveled (Fig. 2a; F_3,1451_ = 8.51, *p* < 0.0001) and stereotypic counts (Fig. 2d; F_3,1451_ = 8.049, *p* < 0.0001), which achieved study-wide significance. Subjects in older age groups traveled significantly shorter distances than those in younger age groups (8–12 mo < 4–5 and 2–3 mo, *p* = 0.0009 and *p* < 0.0001, respectively; 6–7 mo < 2–3 mo, *p* = 0.0012). The 6–7- and 4–5-month-old subjects showed significantly more stereotypic counts than 2–3-month-old subjects (*p* = 0.0007 and *p* < 0.0001, respectively). During the first 5 min, there were trends toward significant effects of Age on the vertical activity (Fig. 2b; F_3,1451_ = 2.25, *p* = 0.0806) and center time (Fig. 2c; F_3,1451_ = 2.38, *p* = 0.0681). The older age groups tended to exhibit reduction in vertical activity and center time compared with the younger age groups (vertical activity, 8–12 mo < 6–7, 4–5, and 2–3 mo, *p* = 0.0504, *p* = 0.0377, and *p* = 0.0103, respectively; center time, 8–12, 6–7, and 4–5 mo < 2–3 mo, *p* = 0.1302, *p* = 0.0859, and *p* = 0.0629, respectively). During the entire 120-min period, there were significant effects of Age on vertical activity (Fig. 2f; F_3,1456_ = 7.77, *p* < 0.0001) and stereotypic counts (Fig. 2h; F_3,1451_ = 5.54, *p* = 0.0009), whereas no significant age effects were found on the distance traveled (Fig. 2e; F_3,1451_ = 1.67, *p* = 0.1721) and center time (Fig. 2g; F_3,1451_ = 1.91, *p* = 0.126). Vertical activity in the 4–5-month-old group was significantly greater than that in the 2–3-month-old group (Fig. 2f; *p* < 0.0001), and stereotypic counts in the 6–7- and 4–5-month-old groups were greater than those in the 2–3-month-old group (Fig. 2h; *p* = 0.003 and *p* = 0.0073, respectively). Overall, the age-dependent decline in locomotor activity during the early testing period in a novel open field environment suggests that aging is associated with increased anxiety-like behavior.

The light/dark transition test is also commonly used to assess anxiety-like behavior. There were significant effects of Age on the distance traveled in the light and dark chambers (Fig. 2i; the light chamber, F_3,1352_ = 11.59, *p* < 0.0001; the dark chamber, F_3,1352_ = 4.46, *p* = 0.004). In the light chamber, subjects in older age groups exhibited significantly shorter distances traveled than those in younger age groups (8–12 mo < 4–5 mo, *p* < 0.0001; 8–12 and 6–7 mo < 2–3 mo, *p* < 0.0001 and *p* = 0.0027, respectively). In the dark chamber, older age subjects also traveled shorter distances than younger age subjects, although *post-hoc* tests revealed that these differences did not survive the Bonferroni correction (8–12, 6–7, and 4–5 mo < 2–3 mo, *p* = 0.0271, *p* = 0.0519, and *p* = 0.0083, respectively). A significant effect of Age was found on the number of transitions between the chambers (Fig. 2j; F_3,1352_ = 35.05, *p* < 0.0001), which achieved study-wide significance. The number of transitions of the older age subjects was significantly lower than those of the younger age subjects (8–12 and 6–7 mo < 4–5 mo, *p* < 0.0001 and *p* = 0.0034, respectively; 8–12, 6–7, and 4–5 mo < 2–3 mo, all comparisons *p* < 0.0001). Regarding latency to enter the light chamber, the oldest age group displayed a longer latency compared with the youngest age group (Fig. 2k; F_3,1352_ = 2.95, *p* = 0.0317; 8–12 mo > 2–3 mo, *p* = 0.0056). There was also significant age effect on the time spent in the light chamber (Fig. 2l; F_3,1352_ = 9.93, *p* < 0.0001), and older age subjects spent less time in the light chamber than younger age subjects (8–12 mo < 4–5 mo, *p* < 0.0001; 8–12 and 6–7 mo < 2–3 mo, *p* < 0.0001 and *p* = 0.0081, respectively). These results of the light/dark transition test imply that locomotor activity decreases and anxiety-like behavior increases from young to middle age.

In the elevated plus maze test that has been widely used for assessing anxiety-like behavior, there was a significant effect of Age on distance traveled (Fig. 2m; F_3,1231_ = 15.11, *p* < 0.0001). This effect reached study-wide significance. Subjects in older age groups traveled significantly shorter distances than those in the youngest age group (8–12, 6–7, and 4–5 mo < 2–3 mo, *p* = 0.0044, *p* < 0.0001, and *p* < 0.0001, respectively). There was also a significant effect of Age on the number of arm entries (Fig. 2n; F_3,1231_ = 19.34, *p* < 0.0001), which attained study-wide significance. Older age subjects showed a significantly lower number of arm entries compared with subjects in the youngest age group (8–12, 6–7, and 4–5 mo < 2–3 mo, *p* = 0.0001, *p* < 0.0001, and *p* < 0.0001, respectively). Significant effects of Age were found on the percentages of entries into open arms (Fig. 2o; F_3,1231_ = 6.84, *p* = 0.0001) and of time on open arms (Fig. 2p; F_3,1231_ = 4.17, *p* = 0.006). The older age groups exhibited a significantly higher percentage of open arm entries than the younger age groups (8–12 mo > 2–3 mo, *p* = 0.0005; 8–12 and 6–7 mo > 4–5 mo, *p* < 0.0001 and *p* = 0.0053, respectively), and 4–5-month-old mice displayed a lower percentage of time spent in open arms than the other age groups (vs. 2–3 mo, *p* = 0.0025; vs. 6–7 mo, *p* = 0.0446; vs. 8–12 mo, *p* = 0.0058). Overall, the age-related decrease in locomotor activity found in this test corroborates the results of the light/dark transition and open field tests. However, the higher percentages of open arm entries and time on open arms in older age groups, which suggest reduced anxiety-like behavior, are seemingly inconsistent with the findings from the other two tests (see Discussion for details).

### Decreased social contacts in older C57BL/6J mice

The social interaction test, in which two unfamiliar mice are placed together in a novel chamber, is used to assess social behavior. There were significant effects of Age on the distance traveled (Fig. 3a; F_3,546_ = 8.48, *p* < 0.0001), number of contacts (Fig. 3b; F_3,546_ = 11.71, *p* < 0.0001), and total duration of active contacts (Fig. 3d; F_3,546_ = 8.58, *p* < 0.0001), all of which reached study-wide significance. Older age subjects traveled significantly shorter distances than subjects in the youngest age group (8–12, 6–7, and 4–5 mo < 2–3 mo, *p* < 0.0001, *p* = 0.0043, and *p* = 0.0055, respectively). The number of contacts in the older age groups was significantly lower than those in the younger age groups (8–12 and 6–7 mo < 2–3 mo, *p* = 0.0002 and *p* < 0.0001, respectively; 8–12 and 6–7 mo < 4–5 mo, *p* = 0.0079 and *p* = 0.0017, respectively). Subjects in older age groups showed a significantly shorter duration of active contacts compared with those in the youngest age group (8–12, 6–7, and 4–5 mo < 2–3 mo, *p* = 0.0056, *p* < 0.0001, and *p* = 0.0022, respectively). There was also significant effect of Age on the total duration of contacts (Fig. 3c; F_3,546_ = 3.93, *p* = 0.0085). The total duration of contacts was shorter in the 6–7-month-old group than in the 2–3-month-old group (*p* = 0.0036). There was no significant age effect in the mean duration per contact (Fig. 3e; F_3,546_ = 0.78, *p* = 0.5044). These results indicate that locomotor activity and social interaction decrease with age in a novel environment.

### Decreased acoustic startle response and increased prepulse inhibition in older C57BL/6J mice

Prepulse inhibition of an acoustic startle response (PPI) is a phenomenon in which a weak prepulse stimulus suppresses the startle response to a loud auditory stimulus, and is measured to assess sensorimotor gating. A test session consists of six trial types, i.e., two types of startle-stimulus-only trials (110 or 120 dB auditory stimulus) and four types for prepulse inhibition trials (74–110, 78–110, 74–120, or 78–120 dB auditory stimulus). In the two types of startle-stimulus-only trials, there were significant effects of Age on the startle response to the loud stimuli (Fig. 4a; 110 dB, F_3,1359_ = 3.56, *p* = 0.0139; 120 dB, F_3, 1359_ = 35.07, *p* < 0.0001), which reached study-wide significance. The startle responses to the 110 dB stimulus in the 8–12-month-old group were significantly lower than those in the other age groups (8–12 mo < 6–7, 4–5, and 2–3 mo, *p* = 0.005, *p* = 0.0011, and *p* = 0.0025, respectively). For the 120 dB stimulus intensity, subjects in older age groups showed significantly lower startle responses than those in younger age groups (8–12 and 6–7 mo < 4–5 mo < 2–3 mo, all comparisons *p* < 0.0001).

In the prepulse inhibition trials, there were significant effects of Age on the prepulse inhibition of the startle response to 78–110, 74–120, and 78–120 dB stimuli (Fig. 4b, F_3,1359_ = 7.75, *p* < 0.0001; F_3,1359_ = 10.03, *p* < 0.0001; F_3,1359_ = 22.94, *p* < 0.0001, respectively). These effects reached study-wide significance, although there was no significant effect of Age on the PPI at 74–110 dB stimulus (F_3,1359_ = 1.86, *p* = 0.1344). The prepulse inhibition of the 78–110 dB stimulus was significantly higher in the 6–7-month-old group than in the 4–5- and 2–3-month-old groups (Fig. 4b; 6–7 mo > 4–5 and 2–3 mo, *p* = 0.0035 and *p* < 0.0001, respectively). Similarly, the prepulse inhibitions at 74–120 dB in the older age groups were significantly higher than those in the youngest age group (6–7 and 4–5 mo > 2–3 mo, *p* < 0.0001 and *p* = 0.0002, respectively). The prepulse inhibitions at 78–120 dB were significantly higher in the 8–12-, 6–7-, and 4–5-month-old groups than in the 2–3-month-old group (*p* = 0.003, *p* < 0.0001, and *p* < 0.0001, respectively) There were no significant differences in the prepulse inhibition between subjects in the 8–12- and 6–7-month-old groups and between those in the 8–12- and 4–5-month-old groups. These results show that the acoustic startle response decreases with aging and that prepulse inhibition of the startle response increases from 2–3 months of age to 6–7 months of age and thereafter appears to stay constant or even decrease.

### Decreased immobility in older C57BL/6J mice

The Porsolt forced swim test, in which a mouse is placed into a cylinder filled with water for 10 min a day for 2 days, is performed to measure immobility time as an index of depression-related behavior. One-way ANOVAs revealed significant effects of Age on immobility time (Fig. 5a: day 1, F_3,1084_ = 3.18, *p* = 0.0234; day 2, F_3,1084_ = 5.64, *p* = 0.0008) and distance traveled (Fig. 5b: day 1, F_3,1084_ = 5.51, *p* = 0.0009; day 2, F_3,1084_ = 1.06, *p* = 0.3671). On day 1, immobility times in older age groups were lower than those in the youngest age group, although these differences did not survive a Bonferroni correction (8–12, 6–7, and 4–5 mo < 2–3 mo, *p* = 0.0255, *p* = 0.03, and *p* = 0.0383, respectively). Similar results were obtained for immobility time on day 2 (8–12 and 4–5 mo < 2–3 mo, *p* = 0.0013 and *p* = 0.0013, respectively). On day 1, the 6–7-month-old subjects swam longer distances than the 4–5- and 2–3-month-old subjects (*p* = 0.0046 and *p* = 0.0002, respectively).

The tail suspension test is another method to evaluate depression-related behavior. There was a significant effect of Age on immobility time in the tail suspension test (Fig. 5c; F_3,396_ = 8.14, *p* < 0.0001), which achieved study-wide significance. Immobility times in the 8–12-month-old group were significantly lower than those in the 4–5- and 2–3-month-old groups (8–12 mo < 4–5 and 2–3 mo, *p* < 0.0001 and *p* < 0.0001, respectively). Overall, the results of these two different types of tests indicate that immobility decreases with age, suggesting an age-related decrease in depression-related behavior.

### Impaired spatial learning and memory in older C57BL/6J mice

To examine age-related changes in spatial learning and memory, behavioral data of the Barnes maze test were analyzed for the first, fifth, and ninth block of two trials during the training session. There were no significant effects of Age on the number of errors (Fig. 6a) in the first or fifth blocks (F_3,349_ = 0.82, *p* = 0.4838; F_3,350_ = 1.44, *p* = 0.2307, respectively); however, a significant effect of Age on the number of errors was found in the ninth block (F_3,349_ = 7.55, *p* < 0.0001), which reached study-wide significance. Subjects in the oldest age group showed a significantly greater number of errors in reaching around the target hole when compared to subjects in the other age groups in the ninth block (8–12 mo > 6–7, 4–5, and 2–3 mo, *p* = 0.0028, *p* = 0.0004, and *p* < 0.0001, respectively). With regard to the latency to reach around the target hole (Fig. 6b), one-way ANOVAs revealed significant effects of Age during the first, fifth, and ninth blocks (F_3,349_ = 2.95, *p* = 0.0327; F_3,350_ = 3.32, *p* = 0.02; F_3,349_ = 3.86, *p* = 0.0097, respectively). In both the first and fifth blocks, 8–12-month-old subjects exhibited longer latencies to reach around the target hole than subjects in the 6–7- and 2–3-month-old groups, although the differences were not significant after a Bonferroni correction (for the first block, 8–12 mo > 6–7 and 2–3 mo, *p* = 0.0278 and *p* = 0.0088, respectively; for the fifth block, 8–12 mo > 6–7 and 2–3 mo, *p* = 0.0131 and *p* = 0.0219, respectively). Similarly, in the ninth block, subjects in the 8–12-month-old group showed longer latencies than 6–7-, 4–5-, and 2–3-month-old groups (*p* = 0.0021, *p* = 0.0195, and *p* = 0.0653, respectively). Regarding distance traveled to reach around the target hole (Fig. 6c), there was no significant Age effect in the first block (F_3,349_ = 1.30, *p* = 0.2742), but significant effects of Age were found in the fifth and ninth blocks (F_3,350_ = 3.50, *p* = 0.0157; F_3,349_ = 10.13, *p* < 0.0001, respectively). Subjects in the 8–12-month-old group traveled longer distance than other age groups in the fifth block (vs. 6–7 mo, *p* = 0.0056; vs. 4–5 mo, *p* = 0.099; vs. 2–3 mo, *p* = 0.0176) and in the ninth block (vs. 6–7 mo, *p* < 0.0001; vs. 4–5 mo, *p* < 0.0001; vs. 2–3 mo, *p* < 0.0001).

At 1 day after the last training session, in the probe test, there was a significant effect of Age on the time spent around the target hole (Fig. 6d; F_3,349_ = 21.86, *p* < 0.0001), which reached study-wide significance. The 8–12-, 6–7-, and 4–5-month-old group subjects spent significantly less time than the 2–3-month-old group subjects (all comparisons *p* < 0.0001), and 8–12-month-old group subjects spent shorter amounts of time around the target hole than 6–7- and 4–5-month-old mice (*p* = 0.0078 and *p* < 0.0001, respectively). It is unlikely that older groups were unable to reach the target hole because of decreased locomotor activity or increased anxiety-like behavior. In fact, there was no significant effect of Age on the number of errors in the probe test (Fig. 6e; F_3,349_ = 2.41, *p* = 0.0667). Additionally, older age mice moved significantly more than mice in the youngest age group over the test period (Fig. 6f: for moving time, F_3,349_ = 38.18, *p* < 0.0001; 8–12 mo > 6–7, 4–5, and 2–3 mo, all comparisons *p* < 0.0001; 4–5 mo > 2–3 mo, *p* < 0.0001; Fig. 6g: for total distance, F_3,349_ = 6.98, *p* = 0.0001; 8–12, 6–7, and 4–5 mo > 2–3 mo, all comparisons *p* < 0.0001), although subjects in the oldest group exhibited reduced moving speed compared with subjects in the younger groups (Fig. 6h; F_3,349_ = 5.39, *p* = 0.0012; 8–12 < 6–7 and 4–5 mo, *p* = 0.0007 and *p* = 0.0008, respectively). These data indicate that spatial learning and memory performance decreases from young adulthood to middle age.

### Reduced contextual and cued fear memory in older C57BL/6J mice

The contextual and cued fear conditioning test is used to assess fear memory. In the conditioning session, freezing behavior and distance traveled during the first 2 min of the session without presentation of the conditioned stimulus (CS, white noise) and unconditioned stimulus (US, footshock) were evaluated to assess baseline activity in the novel environment. In the first 2 min of the session, there was a marginally significant effect of Age on the percentage of freezing (Fig. 7a; F_3,510_ = 2.39, *p* = 0.0678) and there was a significant Age effect on distance traveled (Fig. 7b; F_3,510_ = 5.67, *p* = 0.0008). Older age subjects traveled significantly shorter distances than younger age subjects (8–12 mo < 4–5 and 2–3 mo, *p* = 0.0018 and *p* = 0.0002, respectively). Similarly, during the last 6 min of the conditioning session with CS-US pairings, significant effects of Age were found on the percentage of freezing (F_3,510_ = 12.81, *p* < 0.0001) and distance traveled (F_3,510_ = 4.29, *p* = 0.0053). *Post-hoc* analysis revealed that subjects in the 8–12-month-old group showed significantly greater freezing than those in other age groups (vs. 6–7, 4–5, and 2–3 mo, all comparisons *p* < 0.0001). In addition, the subjects in the 8–12-month-old group traveled shorter distances than subjects in other age groups (vs. 6–7 mo, *p* = 0.0022; vs. 4–5 mo, *p* = 0.0025; vs. 2–3 mo, *p* = 0.0147). During the first US presentation (US1), there was a significant effect of Age on the distance traveled (Fig. 7d; F_3,510_ = 3.30, *p* = 0.0203). Subjects in the 8–12-month-old group traveled shorter distances during the US presentation than 4–5-month-old subjects (*p* = 0.0029). No significant age effects were found in the distance traveled during the second and third US presentations (US2, F_3,510_ = 2.18 *p* = 0.09; US3, F_3,510_ = 0.85, *p* = 0.4691).

In the context test, in which mice are exposed to the same conditioning chamber 24 hr after the conditioning session, no significant effects of Age were found for freezing and distance traveled (F_3,510_ = 0.20, *p* = 0.8971; F_3,510_ = 1.07, *p* = 0.3605, respectively). However, there was a significant effect of Age on the activity suppression ratio as an index of fear [ratio = (distance traveled during the first 2 min in the context test)/(distance traveled during the first 2 min in the conditioning + distance traveled during the first 2 min in the context test), F_3,510_ = 10.50, *p* < 0.0001], which has been used to normalize individual differences in baseline activity [31, 32]. This effect achieved study-wide significance. The activity suppression ratio of the 8–12-month-old group was significantly higher than that of other age groups (Fig. 7c; 8–12 mo > 6–7, 4–5, and 2–3 mo, *p* = 0.0005, *p* < 0.0001, and *p* < 0.0001, respectively). These results suggest that aging is associated with impaired contextual fear memory.

In the cued test following the context test, in which mice were placed in a different shaped chamber with altered context, subjects in the 8–12-, 6–7-, and 4–5-month-old groups exhibited significantly more freezing and traveled shorter distance than 2–3-month-old subjects during the first 3-min period with no CS presentation (the percentage of freezing, F_3,510_ = 11.79, *p* < 0.0001, 8–12, 6–7, and 4–5 mo > 2–3 mo, all *p* < 0.0001; distance traveled, F_3,510_ = 21.20, *p* < 0.0001, 8–12, 6–7, and 4–5 mo < 2–3 mo, all *p* < 0.0001). These differences reached study-wide significance. Similarly, there was a significant effect of Age on the activity suppression ratio (F_3,510_ = 8.13, *p* < 0.0001). The ratio was lower in the 6–7- and 4–5-month-old groups than in the 2–3-month-old group (*p* = 0.0006 and *p* < 0.0001, respectively), and the ratio was lower in the 4–5-month-old group than in the 8–12-month-old group (*p* = 0.0006). Overall, these results suggest that older subjects exhibit increased generalized fear to the altered context. During the last 3 min of the cued test with CS presentation, the freezing percentage of the 8–12-month-old group was significantly lower than those of the 6–7-, 4–5-, and 2–3-month-old groups (F_3,510_ = 8.14, *p* < 0.0001; *p* < 0.0001, *p* = 0.0051, and *p* = 0.0002, respectively). In addition, the distances traveled by the 8–12-month-old mice were significantly greater than those traveled by the 6–7- and 2–3-month-old mice (F_3,510_ = 5.02, *p* = 0.019; vs. 6–7 mo, *p* = 0.0005; vs. 2–3 mo, *p* = 0.0053). Regarding the activity suppression ratio (F_3,510_ = 10.10, *p* < 0.0001), 8–12-month-old mice showed a significantly higher ratio than 6–7-, 4–5-, and 2–3-month-old mice (*p* < 0.0001, *p* < 0.0001, and *p* = 0.0001, respectively). The decreased freezing behavior in the oldest age group suggests impairment of cued fear memory; however, it is possible that the impairment of auditory function in older mice could be responsible for this decreased freezing response to the auditory cue.

## Discussion

In this study, we performed a large-scale analysis of age effects on behavior in C57BL/6J mice by taking advantage of a large amount of behavioral data that we had collected through a behavioral test battery applied to many mutant strains of mice. This battery of behavioral tests was conducted using uniform protocols and apparatuses in our laboratory, although the experimenters and test dates were not necessarily the same. The genetic background of the subjects and their breeding environment were also not uniform because the C57BL/6J mice were obtained from different vendors and laboratories. Therefore, we cannot completely exclude the possibility of potential genetic and environmental factors that may yield behavioral differences between age groups. Nonetheless, the use of a large number of samples may have minimized the distortion of the data by potential influences of these confounding factors, allowing us to detect the subtle but significant effects of age on behavior. Overall, the present results are indicative of age-related changes in physical characteristics, motor function, locomotor activity, anxiety-like behavior, social behavior, prepulse inhibition, depression-related behavior, and learning and memory functions from young adulthood to middle age, although some behavioral differences between age groups did not reach study-wide significance when the highly conservative statistical approach was used.

Our data revealed age-related physiological changes, including gradual increases in body weight and decreases in body temperature, wire hang latency, and rotarod performance, which are generally consistent with previous reports [33–38]. These findings suggest that there are age-related changes from young to middle age in the peripheral and central nervous systems associated with declines in thermoregulation, neuromuscular strength, and motor function in C57BL/6J mice, though the gradual decline in motor function may be explained by an increase in body weight with age. With regard to pain sensitivity, some studies have reported that hot plate latency decreases with age when compared between 4-, 11-, and 24-month-old 129Sv/Ev mice and between 3 and 5- and 19–21-month-old BALB/c mice [39, 40]. A similar finding was reported in 2–28 month old C57BL/6J mice in the tail-withdrawal test [41], suggesting an age-related increase in pain sensitivity. Contrary to these findings, no age-related changes in sensitivity to thermal stimulus were found in our study or a previous study comparing 4- and 28-month-old C57BL/6J mice [6]. Consistent with the results, the present study also showed that there were no substantial differences in distance traveled in response to electric footshock. The discrepancy between the studies might be due to differences in strain, age of testing, and prior test experience, and further study will be required to understand the relationship between age and pain sensitivity in mice.

The open field test is widely used to measure locomotor activity and anxiety-like behavior in a novel environment (for review, see [30]). Previous studies have reported that older C57BL/6 mice showed reductions in the distance traveled and center time when compared with younger mice [42–44]. Similarly, the present results indicated an age-related decrease from young to middle age in the distance traveled during the first 5 min of the open field test when anxiety-like behavior has been generally assessed in the test [30]. It is unlikely that the decreased distance traveled is due to age-related declines in muscular strength and motor function as shown in the wire hang and rotarod tests, since the total distance traveled during the entire test period did not significantly differ between age groups, which suggests no reduction in general activity with age. In agreement with the findings of the open field test, older mice traveled shorter distances than younger mice in the light/dark transition, elevated plus maze, and social interaction tests, which have also been used to assess anxiety-like behavior [45–48], whereas no reduction in distance traveled in older mice was found in the dark chamber of the light/dark transition apparatus that is considered to be less anxiogenic environment. Together, these findings suggest that the decreased locomotor activity in older mice during the early period may be due to an increased emotional response to a novel environment, and not age-related muscle weakness and motor dysfunction. Interestingly, older mice tended to spend less time in the center area than younger mice during the first 5 min of the open field test. In addition, older mice spent less time in the light chamber and exhibited longer latency to first entry into the light chamber compared with younger mice in the light/dark transition test. Together, these results support the conclusion that anxiety-like behavior increases with age.

In contrast, older mice showed increased percentages of open arm entries and time spent in open arms in the elevated plus maze test, which could be interpreted as a decrease in anxiety-like behavior. Such conflicting results from the two different tests have been reported in several inbred and knockout mouse studies [49–52]. These reports suggest that the behavioral indices of each test may reflect different aspects of anxiety-like behavior, as has been confirmed by principal component analyses [53, 54]. Some researchers have speculated that the increased exploration of open arms may reflect an increased panic-like escape response to stress and/or a higher level of anxiety [50–52, 55], which may be partially supported by the finding that mice showing increased open arm exploration exhibited increased stress response or a higher plasma corticosterone level [52]. Aged animals showed higher corticosterone levels than younger animals after exposure to a novel environment or a sudden noise [56–58]. These findings suggest that anxiety- and panic-like behaviors in response to a novel/stressful environment increase with age from young adulthood to middle age. However, the developmental processes of anxiety- or panic-like behaviors need to be further investigated since previous studies have reported some inconsistent findings on age-related changes in the anxiety-like behaviors assessed in these tests [42, 43, 59–62].

Reduced locomotor activity and social contacts in older mice were observed in the social interaction test. Our results are consistent with previous reports showing that middle-aged and aged animals exhibited decreased social behavior than their young counterparts [39, 59, 63, 64]. In the social interaction test, the reduction of locomotor activity and social contacts may reflect increased anxiety (for review, see [47]). The decreased social behavior in older mice seems to agree with the findings obtained from the open field and light/dark transition tests. These findings suggest that aging from young adulthood to middle age is associated with decreases in social motivation to approach and social investigation accompanied by increased anxiety in response to a novel social environment.

Our results revealed that the startle response to a 110 dB stimulus does not change from approximately 2–7 months of age, and decreases thereafter. Additionally, the startle response to a 120 dB stimulus is the highest at 2–3 months of age and then gradually decreases with age. These findings suggest that younger mice are more sensitive to the auditory stimuli than older mice, indicating that there is an age-related decrease in the startle response to a loud noise. C57BL/6J mice show cochlear degeneration and hearing loss with advancing age [65, 66]. Therefore, the age-related decline in the acoustic responses may be partly attributed to age-related hearing impairments. Prepulse inhibition of the startle response in C57BL/6J mice was found to increase from approximately 2–7 months of age and tend to decrease thereafter. Similar inverted U-shaped changes with age have been reported in previous studies [66, 67]. Given that there was little to no correlation between startle response and percentage of prepulse inhibition (correlation coefficients ranging from *r* = −0.192 to *r* = 0.154), the age-related changes in prepulse inhibition seems not to be simply related to the age-related decrease in startle response or hearing ability. Although it remains unclear why there is an inverted U-shaped relation between age and prepulse inhibition, these findings suggest that auditory information processing in the central nervous system changes from young adulthood to middle age.

The effect of age on depression-related behavior remains controversial because previous studies have shown inconsistent findings on depression-related behavior in rats and mice [60, 62, 68–72]. Some studies in mice have reported that young, middle-aged, and aged mice showed no differences in immobility in the forced swim and tail suspension tests [60, 68, 69, 72]. However, our data indicated that immobility decreases from young adulthood to middle age in the two tests. One of the reasons for the age-related differences found in our study is that the sample size was large enough to detect statistically significant differences. Another possible explanation for the discrepancy between studies is that there might be the differences in animal species, strain, behavioral procedures, and prior experience with stress. Prior test experience and stress can alter subsequent behavioral responses [24, 26, 73–77]. In our study, mice were subjected to a number of behavioral tests before the Porsolt forced swim and tail suspension tests. This test history may have contributed to the age-related differences in depression-related behavior, although further study will be needed to clarify the precise effects of age coupled with prior test experience on depression-related behavior.

The effect of age on spatial memory has been intensively studied using the Barnes maze task [78] and the Morris water maze task (for review, see [79]) in rats and mice. The spatial memory deficit in the water maze task has been found in C57BL/6 mice aged approximately above 12 months [80–85]. However, since this task requires animals to have the ability and motivation to swim, the group differences in memory performance might be due to differences in swimming ability [86]. Furthermore, the task paradigm is a stressful situation, which can affect memory performance. In contrast, the Barnes maze task is a dry-land task that is less stressful and may be a more appropriate task for mice [87, 88], in which memory performance has not been systematically evaluated across the entire lifespan [79]. Our data indicated that 8–12-month-old mice exhibited an increase in the number of errors, latency to approach the target, and distance traveled to the target hole when compared with younger mice during training session. These results are consistent with a previous report [89], indicating that spatial learning and memory functions decline from 12 months of age in C57BL/6J mice. The present results of the probe test further indicate that spatial memory deficits can be observed after 4–5 months of age. It is of interest to examine the effect of age on other spatial learning and memory tasks, such as the Morris water maze and eight-arm radial maze tasks.

A number of studies have reported little or no age-related differences in the contextual and cued fear memory performance in rats and mice (e.g., [90–95]; for review, see [79, 96]), whereas some studies have found age-related deficits in contextual memory when comparing young (3–6-months-old) and aged (16–18-months-old) C57BL/6 mice [97, 98]. Our study showed that activity suppression ratio in the context test increased at 8 months of age, suggesting that contextual fear memory deficit can occur after middle age in C57BL/6 mice. In addition, we found decreased freezing and increased activity suppression ratio in the altered context during CS presentation in 8–12-months old mice, showing deficit in cued fear memory. Furthermore, the present study found an increase in freezing behavior in 4–12-month-old mice compared with 2–3-month-old mice when the CS was not presented in the altered context, suggesting an age-related increase in generalized fear/anxiety or age-related deficits in the ability to discriminate between contexts, or spatial pattern separation.

Overall, the present study reveals that there are age-related changes in various behaviors from young adulthood to middle age. Some of the behavioral changes were observed at the early stage of adulthood, the occurrences of which depended on the types of behavioral tests implemented. For example, decreased locomotor activity occurred between 2–3 months of age and 4–5 months of age, as shown in the number of transitions in the light/dark transition test and in the distance traveled in the elevated plus maze and social interaction tests in a novel environment, whereas such marked changes were not found in the open field test and in the dark chamber of the light/dark transition test. Similarly, there was a gradual decrease in rotarod performance and acoustic startle response after 2–3 months of age. For the Barnes maze test, spatial memory performance decreased after 2–3 months of age, though there were no significant differences in performance between subjects of 4–5 and 6–7 months old, suggesting that spatial memory function was stable during 4–7 months of age. In contrast, prepulse inhibition of the startle response increased until 6–7 months of age, and then appeared to decrease after middle age. The pain sensitivity to thermal stimulus and electric footshock was stable from young adulthood to middle age. These findings show that the age of the subjects is one of the possible confounding factors influencing behavioral outcomes and therefore, needs to be taken into account in designing and conducting behavioral tests involving a cohort of mice.

Aging is associated with various behavioral changes that are mediated by brain structures and networks [2, 99, 100]. The age-related changes in behaviors have usually been found by comparing young and aged animals in behavioral tests. Our large-scale analysis using a number of behavioral data from mice subjected to a behavioral test battery demonstrated that almost all the behaviors examined, including locomotor activity, anxiety-like behavior, social behavior, startle response, depression-related behavior, spatial learning and memory, and associative fear memory, gradually change from young adulthood to middle age. In this study, we used the data of C57BL/6J mice with, which are the most widely used inbred strain for creating genetically engineered mice and studying mouse behavioral phenotypes. These findings will provide further opportunities to understand the neurobiological processes underlying brain function and behavior that are changeable with age from early to middle life.

## Conclusions

In the present study, our large-scale analysis of the effects of age on behavior demonstrates that various behaviors change from young adulthood to middle age in C57BL/6J mice. Our findings provide insights into understanding the developmental process and the underlying mechanisms of brain and behavior in C57BL/6J mice. In addition, our study showed that relatively narrow age differences can produce great variability in behavior during adulthood. Age is therefore one of the critical factors influencing behaviors that should be carefully considered when designing behavioral experiments and interpreting behavioral differences that could be induced by experimental manipulations.

## Methods

### Animals and experimental design

Genetically engineered mice and their wild-type control mice were transported from the animal facilities of other laboratories or vendors to our laboratory, and subjected to a behavioral test battery. Behavioral data of up to 1739 wild-type control male mice that we have collected from the behavioral analysis of 61 strains of genetically engineered mice with a C57BL/6J genetic background were used for analysis in this study. We did not exclude any behavioral data except for some specific cases in which animals fell from a testing apparatus or data were not recorded due to some technical problems. More than 90 % of the mice were backcrossed at least six times (and more than 95 % of the mice used were backcrossed at least five times) with C57BL/6J mice. The wild-type control mice, which were derived from JAX C57BL/6J strain or C57BL/6J substrains (6JJcl or 6JJmsSlc) maintained in Japan, were regarded as “C57BL/6J” mice. They were housed in plastic cages with sterilized PaperClean Bedding (Japan SLC) under a 12-hr light/dark cycle (lights on at 7:00 am) with access to food (CRF-1, Oriental Yeast Co., Ltd.) and water *ad libitum* in our animal facilities. Behavioral testing was performed between 9:00 a.m. and 6:00 p.m. Of the mice, 1.3, 2.3, 6.7, 80.1, 8.9, and 0.7 % were housed with 1, 2, 3, 4, 5, and 6 animals per cage, respectively, at the beginning of the test battery. The mice were generally tested in the following order; general health and neurological screening, light/dark transition, open field, elevated plus maze, hot plate, social interaction, rotarod, startle response/prepulse inhibition, Porsolt forced swim, Barnes maze, contextual and cued fear conditioning, and tail suspension tests. The interval between tests was at least 1 day. More than 75 % of the mice were subjected to the behavioral tests in accordance with the order of the test battery, although in some strains of mice, several tests were omitted from the test battery. For the remaining mice, several tests were performed while changing the order of the test and/or were omitted from the test battery. After the tests, all apparatus were cleaned with super hypochlorous water and 70 % ethanol to prevent a bias due to olfactory cues. The information about each mouse and behavioral data used in this study are open on a public database “Mouse Phenotype Database” (URL: http://www.mouse-phenotype.org/). All behavioral testing procedures were approved by the Animal Care and Use Committee of Kyoto University Graduate School of Medicine and National Institute for Physiological Sciences in Japan.

### General health and neurological screen

The general health check and neurological screen was conducted as previously described [101–103]. Body weight and rectal temperature were measured. Neuromuscular strength was assessed using the grip strength and wire hang tests. A grip strength meter (O’Hara & Co., Tokyo, Japan) was used to assess forelimb grip strength. Mice were lifted and held by their tail so that their forepaws could grasp a wire grid. The mice were then gently pulled backward by the tail until they released the grid. The peak force applied by the forelimbs of the mouse was recorded in Newtons (N). Each mouse was tested three times, and the largest value was used for statistical analysis. In the wire hang test, the mouse was placed on a wire mesh that was then inverted, and the latency to fall from the wire was recorded with a 60 s cut-off time.

### Light/dark transition test

The light/dark transition test, originally developed by Crawley and colleagues [104], was performed as previously described [105]. The apparatus consisted of a cage (21 × 42 × 25 cm) divided into two sections of equal size by a partition with a door (O’Hara & Co., Tokyo, Japan). One chamber was brightly illuminated (390 lux), whereas the other chamber was dark (2 lux). Mice were placed into the dark chamber and allowed to move freely between the chambers with the door open for 10 min. The total number of transitions between chambers, time spent in each chamber (s), latency to first enter the light chamber (s), and distance traveled in each chamber (cm) were recorded automatically by ImageLD software (see Section, “Image analysis”).

### Open field test

The open field test was used to evaluate locomotor activity and emotional response [30]. The apparatus was a transparent square cage (42 × 42 × 30 cm; Accuscan Instruments, Columbus, OH, USA). The center of the floor was illuminated at 100 lux. Each mouse was placed in the open field apparatus and recorded for 120 min. Total distance traveled (cm), vertical activity (rearing measured by counting the number of photobeam interruptions), time spent in the center area (20 × 20 cm), and the beam-break counts for stereotyped behaviors were measured.

### Elevated plus maze test

The elevated plus maze test was conducted as previously described [106]. The elevated plus maze consisted of two open arms (25 × 5 cm, with 3-mm-high ledges) and two closed arms (25 × 5 cm, with 15-cm-high transparent walls) of the same size (O’Hara & Co., Tokyo, Japan). The arms and central square were made of white plastic plates and were elevated to a height of 55 cm above the floor. The arms of the same type were arranged at opposite sides to each other. The center of the maze was illuminated at 100 lux. Each mouse was placed in the central square of the maze (5 × 5 cm), facing one of the closed arms, and was recorded for 10 min. The distance traveled (cm), number of total entries into arms, percentage of entries into open arms, and percentage of time spent in open arms were calculated automatically using ImageEP software (see Section, “Image analysis”).

### Hot plate test

The hot plate test was performed to evaluate sensitivity to a painful stimulus. Mice were placed on a 55.0 ± 0.3 °C hot plate (Columbus Instruments, Columbus, OH, USA), and latency to the first paw response (s) was recorded with a 15 s cut-off time. The paw response was defined as either a foot shake or a paw lick.

### Social interaction test in a novel environment

Social interaction test [107, 108] was conducted as previously described [51]. Two mice of same genotype that were previously housed in different cages, were placed into a box together (40 × 40 × 30 cm; O’Hara & Co., Tokyo, Japan) and were allowed to explore freely for 10 min. Mouse behavior was analyzed automatically using ImageSI software (see Section, “Image analysis”). The total duration of contacts (s), number of contacts, total duration of active contacts (s), mean duration per contact, and total distance traveled (cm) were measured. The active contact was defined as follows: images were captured at three frames per second, and distance traveled between two successive frames was calculated for each mouse. If the two mice contacted each other and the distance traveled by either mouse was 5 cm and more, the behavior was considered an “active contact.”

### Startle response/prepulse inhibition test

A startle reflex measurement system (O’Hara & Co., Tokyo, Japan) was used to measure startle response to a loud noise and prepulse inhibition of the startle response. A test session began by placing a mouse in a plastic cylinder where it was left undisturbed for 10 min. White noise (40 ms) was used as the startle stimulus for all trial types. The startle response was recorded for 400 ms starting with the onset of the startle stimulus. The background noise level was 70 dB. The peak startle amplitude was used as a dependent variable. A test session consisted of six trial types (e.g., two types of startle-stimulus-only trials, and four types for prepulse inhibition trials). The intensity of the startle stimulus was either 110 or 120 dB. The prepulse sound was presented 100 ms before the onset of the startle stimulus, and its intensity was 74 or 78 dB (20 ms). Four combinations of prepulse and startle stimuli were used (74–110, 78–110, 74–120, and 78–120 dB). Six blocks of the six trial types were presented in a pseudorandom order such that each trial type was presented once within a block. The average inter-trial interval was 15 s (range: 10–20 s).

### Porsolt forced swim test

The Porsolt forced swim test, developed by Porsolt et al. [109], was performed as previously described [102, 110, 111]. The apparatus consisted of four plastic cylinders (20 × 10 cm; O’Hara & Co., Tokyo, Japan). The cylinders were filled with water (approximately 23 °C) to a height of 7.5 cm. Mice were placed into the cylinders, and the immobility and distance traveled were recorded over a 10-min test period. Images were captured, and for each pair of successive frames, the amount of area (pixels) within which the mouse moved was measured. When the amount of area was below a certain threshold, mouse behavior was judged as “immobile”. When the amount of area equaled or exceeded the threshold, the mouse was considered as “moving”. The optimal threshold by which to judge was determined by adjusting it to the amount of immobility measured by human observation. Immobility lasting for less than 2 s was not included in the analysis. Data acquisition and analysis were performed automatically using ImagePS software (see Section, “Image analysis”).

### Barnes maze test

The Barnes circular maze task, developed by Barnes [78], was conducted on a white circular surface (1.0 m in diameter, with 12 holes equally spaced around the perimeter; O’hara & Co., Tokyo, Japan). The circular open field was elevated 75 cm from the floor. A black Plexiglas escape box (17 × 13 × 7 cm), which had paper cage bedding on its bottom, was located under one of the holes. The hole above the escape box represented the target, analogous to the hidden platform in the Morris water maze task. The location of the target was consistent for a given mouse but randomized across mice. The maze was rotated daily, with the spatial location of the target unchanged with respect to the distal visual room cues, to prevent a bias based on olfactory or proximal cues within the maze. One to four trials per day were conducted as training sessions. The number of errors, latency to reach the target (s), and distance traveled to reach the target (cm) were automatically calculated by ImageBM software (see Section, “Image analysis”). After the last training session, a probe test was conducted without the escape box for 3 min, to confirm that this spatial task was acquired based on navigation by distal environment cues. The time spent around each hole, number of errors, moving time (s), distance traveled (cm), and moving speed (cm/s) were recorded using ImageBM software.

### Contextual and cued fear conditioning test

Contextual and cued fear conditioning test was performed as previously described [112]. In brief, each mouse was placed in a transparent acrylic chamber (33 × 25 × 28 cm) with a stainless-steel grid floor (0.2 cm diameter, spaced 0.5 cm apart; O’Hara & Co., Tokyo, Japan) and was allowed to explore freely for 2 min. Subsequently, a 55 dB white noise, which served as the conditioned stimulus (CS), was presented for 30 s. During the last 2 s of CS presentation, a mild footshock (0.3 mA, 2 s), which served as the unconditioned stimulus (US), was presented. Two more CS-US pairings were presented with a 2-min inter-stimulus interval. Twenty-four hours after the conditioning session, a context test was conducted in the same chamber. A cued test with altered context was then performed in a triangular chamber (33 × 29 × 32 cm) made of white opaque plastic, which was located in a different room. In each test, freezing percentage and distance traveled were calculated automatically using ImageFZ software (see Section, “Image analysis”).

### Tail suspension test

Tail suspension test, developed by Steru et al. [113], was performed as described previously [114]. Each mouse was suspended 30 cm above the floor by the tail in a white plastic chamber (31 × 41 × 41 cm) (O’Hara & Co., Tokyo, Japan). The behavior was recorded for 10 min. Images were captured through a video camera, and immobility was measured. Similar to the Porsolt forced swim test, immobility was judged by ImageTS software (see Section, “Image analysis”) according to a certain threshold. Immobility lasting for less than a 2 s was not included in the analysis.

### Image analysis

The application software used for the behavioral experiments (ImageLD, EP, SI, PS, BM, FZ, and TS) were based on the public domain NIH Image program (developed at the U.S. National Institutes of Health and available at http://rsb.info.nih.gov/nih-image/) and ImageJ program (http://rsb.info.nih.gov/ij/), which were modified for each test by Tsuyoshi Miyakawa.

### Statistical analysis

Statistical analysis was conducted using SAS University Edition (SAS Institute, Cary, NC). Data were analyzed using a one-way ANOVA. We set the “study-wide significance” level to *p* < 0.05/69 = 0.000724 by Bonferroni correction based on 69 behavioral measures used in the test battery. “Nominal significance” was defined as the one that achieved a statistical significance in an index (*p* < 0.05) but did not survive the correction. The *post-hoc* multiple comparisons were further performed using Fisher’s PLSD with Bonferroni correction (for the study wide significance, *p* < 0.000724/6 = 0.00012; for the nominal significance, *p* < 0.05/6 = 0.008333). Values in graphs are expressed as mean ± SEM.

## Additional files

Additional file 1: Table S1. Statistical analyses of behavioral data. (XLS 40 kb) Additional file 2: Figure S1. Correlation between rotarod latency and body weight. Correlations between rotarod latency (s) and body weight (g) in (A) all age groups, (B) 2–3-month-old group, (C) 4–5-months-old group, (D) 6–7-months-old group, and (E) 8–12-months-old group. (F) Correlation between rotarod latency (s) and body weight (g) in each age group with body weights ranging from 27.5 to 32.5 g and (G) the mean rotarod latency. (EPS 2206 kb)

## References

[1] Bishop NA, Lu T, Yankner BA (2010). Neural mechanisms of ageing and cognitive decline. Nature.

[2] Burke SN, Barnes CA (2006). Neural plasticity in the ageing brain. Nat Rev Neurosci.

[3] Mattson MP, Chan SL, Duan W (2002). Modification of brain aging and neurodegenerative disorders by genes, diet, and behavior. Physiol Rev.

[4] Ammassari-Teule M, Fagioli S, Rossi-Arnaud C (1994). Radial maze performance and open-field behaviours in aged C57BL/6 mice: further evidence for preserved cognitive abilities during senescence. Physiol Behav.

[5] Brennan MJ, Dallob A, Friedman E (1981). Involvement of hippocampal serotonergic activity in age-related changes in exploratory behavior. Neurobiol Aging.

[6] Fahlström A, Zeberg H, Ulfhake B (2012). Changes in behaviors of male C57BL/6J mice across adult life span and effects of dietary restriction. Age.

[7] Flood JF, Morley JE (1990). Pharmacological enhancement of long-term memory retention in old mice. J Gerontol.

[8] Fordyce DE, Whner JM (1993). Effects of aging on spatial learning and hippocampal protein kinase C in mice. Neurobiol Aging.

[9] Forster MJ, Dubey A, Dawson KM, Stutts WA, Lal H, Sohal RS (1996). Age-related losses of cognitive function and motor skills in mice are associated with oxidative protein damage in the brain. Proc Natl Acad Sci.

[10] Frick KM, Burlingame LA, Arters JA, Berger-Sweeney J (1999). Reference memory, anxiety and estrous cyclicity in C57BL/6NIA mice are affected by age and sex. Neuroscience.

[11] Hengemihle JM, Long JM, Betkey J, Jucker M, Ingram DK (1999). Age-related psychomotor and spatial learning deficits in 129/SvJ mice. Neurobiol Aging.

[12] Ingram DK, London ED, Reynolds MA, Waller SB, Goodrick CL (1981). Differential effects of age on motor performance in two mouse strains. Neurobiol Aging.

[13] Sprott RL, Eleftheriou BE (1974). Open-field behavior in aging inbred mice. Gerontology.

[14] Chesselet MF, Richter F (2011). Modelling of Parkinson’s disease in mice. Lancet Neurol.

[15] Hall AM, Roberson ED (2012). Mouse models of Alzheimer’s disease. Brain Res Bull.

[16] Crawley JN (2008). Behavioral phenotyping strategies for mutant mice. Neuron.

[17] Crawley JN, Paylor R (1997). A proposed test battery and constellations of specific behavioral paradigms to investigate the behavioral phenotypes of transgenic and knockout mice. Horm Behav.

[18] Karl T, Pabst R, von Hörsten S (2003). Behavioral phenotyping of mice in pharmacological and toxicological research. Exp Toxicol Pathol.

[19] Powell CM, Miyakawa T (2006). Schizophrenia-relevant behavioral testing in rodent models: a uniquely human disorder?. Biol Psychiatry.

[20] Rogers DC, Fisher EMC, Brown SDM, Peters J, Hunter AJ, Martin JE (1997). Behavioral and functional analysis of mouse phenotype: SHIRPA, a proposed protocol for comprehensive phenotype assessment. Mamm Genome.

[21] Takao K, Yamasaki N, Miyakawa T (2007). Impact of brain-behavior phenotypying of genetically-engineered mice on research of neuropsychiatric disorders. Neurosci Res.

[22] Crabbe JC, Wahlsten D, Dudek BC (1999). Genetics of mouse behavior: interactions with laboratory environment. Science.

[23] Holmes A, Iles JP, Mayell SJ, Rodgers RJ (2001). Prior test experience compromises the anxiolytic efficacy of chlordiazepoxide in the mouse light/dark exploration test. Behav Brain Res.

[24] McIlwain KL, Merriweather MY, Yuva-Paylor LA, Paylor R (2001). The use of behavioral test batteries: effects of training history. Physiol Behav.

[25] Wahlsten D, Metten P, Phillips TJ, Boehm SL, Burkhart-Kasch S, Dorow J (2003). Different data from different labs: lessons from studies of gene–environment interaction. J Neurobiol.

[26] Voikar V, Vasar E, Rauvala H (2004). Behavioral alterations induced by repeated testing in C57BL/6J and 129S2/Sv mice: implications for phenotyping screens. Genes Brain Behav.

[27] Matsuo N, Takao K, Nakanishi K, Yamasaki N, Tanda K, Miyakawa T (2010). Behavioral profiles of three C57BL/6 substrains. Front Behav Neurosci.

[28] Holmes A, Yang RJ, Murphy DL, Crawley JN (2002). Evaluation of antidepressant-related behavioral responses in mice lacking the serotonin transporter. Neuropsychopharmacology.

[29] McFadyen MP, Kusek G, Bolivar VJ, Flaherty L (2003). Differences among eight inbred strains of mice in motor ability and motor learning on a rotorod. Genes Brain Behav.

[30] Prut L, Belzung C (2003). The open field as a paradigm to measure the effects of drugs on anxiety-like behaviors: a review. Eur J Pharmacol.

[31] Frankland PW, O’Brien C, Ohno M, Kirkwood A, Silva AJ (2001). α-CaMKII-dependent plasticity in the cortex is required for permanent memory. Nature.

[32] Anagnostaras SG, Wood SC, Shuman T, Cai DJ, LeDuc AD, Zurn KR (2010). Automated assessment of pavlovian conditioned freezing and shock reactivity in mice using the video freeze system. Front Behav Neurosci.

[33] Butterfield NN, Graf P, Ries CR, MacLeod BA (2004). The effect of repeated isoflurane anesthesia on spatial and psychomotor performance in young and aged mice. Anesth Analg.

[34] Halloran BP, Ferguson VL, Simske SJ, Burghardt A, Venton LL, Majumdar S (2002). Changes in bone structure and mass with advancing age in the male C57BL/6J mouse. J Bone Miner Res.

[35] Talan M (1984). Body temperature of C57BL/6J mice with age. Exp Gerontol.

[36] Talan MI, Engel BT (1986). Temporal decrease of body temperature in middle-aged C57BL/6J mice. J Gerontol.

[37] Shukitt–Hale B, Smith DE, Meydani M, Joseph JA (1999). The effects of dietary antioxidants on psychomotor performance in aged mice. Exp Gerontol.

[38] Serradj N, Jamon M (2007). Age-related changes in the motricity of the inbred mice strains 129/sv and C57BL/6j. Behav Brain Res.

[39] Berry A, Capone F, Giorgio M, Pelicci PG, De Kloet ER, Alleva E (2007). Deletion of the life span determinant p66^Shc^ prevents age-dependent increases in emotionality and pain sensitivity in mice. Exp Gerontol.

[40] Matzel LD, Grossman H, Light K, Townsend D, Kolata S (2008). Age-related declines in general cognitive abilities of Balb/C mice are associated with disparities in working memory, body weight, and general activity. Learn Mem.

[41] Webster GW, Shuster L, Eleftheriou BE (1976). Morphine analgesia in mice of different ages. Exp Aging Res.

[42] Benice TS, Rizk A, Kohama S, Pfankuch T, Raber J (2006). Sex-differences in age-related cognitive decline in C57BL/6J mice associated with increased brain microtubule-associated protein 2 and synaptophysin immunoreactivity. Neuroscience.

[43] Davis MJ, Haley T, Duvoisin RM, Raber J (2012). Measures of anxiety, sensorimotor function, and memory in male and female mGluR4^−/−^ mice. Behav Brain Res.

[44] Lalonde R, Strazielle C (2009). Exploratory activity and motor coordination in old versus middle-aged C57BL/6J mice. Arch Gerontol Geriatr.

[45] Belzung C, Griebel G (2001). Measuring normal and pathological anxiety-like behaviour in mice: a review. Behav Brain Res.

[46] Bourin M, Hascoet M (2003). The mouse light/dark box test. Eur J Pharmacol.

[47] File SE, Seth P (2003). A review of 25 years of the social interaction test. Eur J Pharmacol.

[48] Walf AA, Frye CA (2007). The use of the elevated plus maze as an assay of anxiety-related behavior in rodents. Nat Protoc.

[49] Griebel G, Belzung C, Perrault G, Sanger DJ (2000). Differences in anxiety-related behaviours and in sensitivity to diazepam in inbred and outbred strains of mice. Psychopharmacology (Berl).

[50] Hattori S, Takao K, Tanda K, Toyama K, Shintani N, Baba A (2011). Comprehensive behavioral analysis of pituitary adenylate cyclase-activating polypeptide (PACAP) knockout mice. Front Behav Neurosci.

[51] Miyakawa T, Leiter LM, Gerber DJ, Gainetdinov RR, Sotnikova TD, Zeng H (2003). Conditional calcineurin knockout mice exhibit multiple abnormal behaviors related to schizophrenia. Proc Natl Acad Sci.

[52] Takao K, Kobayashi K, Hagihara H, Ohira K, Shoji H, Hattori S (2013). Deficiency of Schnurri-2, an MHC enhancer binding protein, induces mild chronic inflammation in the brain and confers molecular, neuronal, and behavioral phenotypes related to schizophrenia. Neuropsychopharmacology.

[53] Belzung C, Le Pape G (1994). Comparison of different behavioral test situations used in psychopharmacology for measurement of anxiety. Physiol Behav.

[54] Milner LC, Crabbe JC (2008). Three murine anxiety models: results from multiple inbred strain comparisons. Genes Brain Behav.

[55] Holmes A, Parmigiani S, Ferrari PF, Palanza P, Rodgers RJ (2000). Behavioral profile of wild mice in the elevated plus-maze test for anxiety. Physiol Behav.

[56] Algeri S, Biagini L, Garofalo P, Marconi M, Pitsikas N, Sacchetti G (1990). Adrenocortical and central monoaminergic system responses to different stressful situations in young and senescent rats. Psychobiol Stress.

[57] Herman JP, Larson BR, Speert DB, Seasholtz AF (2001). Hypothalamo-pituitary adrenocortical dysregulation in aging F344/Brown-Norway F1 hybrid rats. Neurobiol Aging.

[58] Küçük A, Gölgeli A, Saraymen R, Koç N (2008). Effects of age and anxiety on learning and memory. Behav Brain Res.

[59] Francia N, Cirulli F, Chiarotti F, Antonelli A, Aloe L, Alleva E (2006). Spatial memory deficits in middle-aged mice correlate with lower exploratory activity and a subordinate status: role of hippocampal neurotrophins. Eur J Neurosci.

[60] Malatynska E, Steinbusch HW, Redkozubova O, Bolkunov A, Kubatiev A, Yeritsyan NB (2012). Anhedonic-like traits and lack of affective deficits in 18-month-old C57BL/6 mice: implications for modeling elderly depression. Exp Gerontol.

[61] Bedrosian TA, Herring KL, Weil ZM, Nelson RJ (2011). Altered temporal patterns of anxiety in aged and amyloid precursor protein (APP) transgenic mice. Proc Natl Acad Sci.

[62] Schulz D, Huston JP, Buddenberg T, Topic B (2007). “Despair” induced by extinction trials in the water maze: Relationship with measures of anxiety in aged and adult rats. Neurobiol Learn Mem.

[63] Salchner P, Lubec G, Singewald N (2004). Decreased social interaction in aged rats may not reflect changes in anxiety-related behaviour. Behav Brain Res.

[64] Shoji H, Mizoguchi K (2011). Aging-related changes in the effects of social isolation on social behavior in rats. Physiol Behav.

[65] Keithley EM, Canto C, Zheng QY, Fischel-Ghodsian N, Johnson KR (2004). Age-related hearing loss and the *ahl* locus in mice. Hear Res.

[66] Ouagazzal AM, Reiss D, Romand R (2006). Effects of age-related hearing loss on startle reflex and prepulse inhibition in mice on pure and mixed C57BL and 129 genetic background. Behav Brain Res.

[67] Willott JF, Carlson S, Chen H (1994). Prepulse inhibition of the startle response in mice: relationship to hearing loss and auditory system plasticity. Behav Neurosci.

[68] David DJP, Bourin M, Hascoët M, Colombel MC, Baker GB, Jolliet P (2001). Comparison of antidepressant activity in 4-and 40-week-old male mice in the forced swimming test: involvement of 5-HT1A and 5-HT1B receptors in old mice. Psychopharmacology (Berl).

[69] Godbout JP, Moreau M, Lestage J, Chen J, Sparkman NL, O’Connor J (2008). Aging exacerbates depressive-like behavior in mice in response to activation of the peripheral innate immune system. Neuropsychopharmacology.

[70] Kasckow JW, Segar TM, Xiao C, Furay AR, Evanson NK, Ostrander MM (2005). Stability of neuroendocrine and behavioral responsiveness in aging Fischer 344/Brown-Norway hybrid rats. Endocrinology.

[71] Moretti M, de Souza AG, de Chaves G, de Andrade VM, Romao PRT, Gavioli EC (2011). Emotional behavior in middle-aged rats: implications for geriatric psychopathologies. Physiol Behav.

[72] Ohashi S, Mori A, Kurihara N, Mitsumoto Y, Nakai M (2006). Age-related severity of dopaminergic neurodegeneration to MPTP neurotoxicity causes motor dysfunction in C57BL/6 mice. Neurosci Lett.

[73] Bertoglio LJ, Carobrez AP (2000). Previous maze experience required to increase open arms avoidance in rats submitted to the elevated plus-maze model of anxiety. Behav Brain Res.

[74] Brett RR, Pratt JA (1990). Chronic handling modifies the anxiolytic effect of diazepam in the elevated plus-maze. Eur J Pharmacol.

[75] Korte SM, De Boer SF (2003). A robust animal model of state anxiety: fear-potentiated behaviour in the elevated plus-maze. Eur J Pharmacol.

[76] Schmitt U, Hiemke C (1998). Strain differences in open-field and elevated plus-maze behavior of rats without and with pretest handling. Pharmacol Biochem Behav.

[77] Swiergiel AH, Leskov IL, Dunn AJ (2008). Effects of chronic and acute stressors and CRF on depression-like behavior in mice. Behav Brain Res.

[78] Barnes CA (1979). Memory deficits associated with senescence: a neurophysiological and behavioral study in the rat. J Comp Physiol Psychol.

[79] Kennard JA, Woodruff-Pak DS (2011). Age sensitivity of behavioral tests and brain substrates of normal aging in mice. Front Aging Neurosci.

[80] Bellush LL, Wright AM, Walker JP, Kopchick J, Colvin RA (1996). Caloric restriction and spatial learning in old mice. Physiol Behav.

[81] Gower AJ, Lamberty Y (1993). The aged mouse as a model of cognitive decline with special emphasis on studies in NMRI mice. Behav Brain Res.

[82] Harburger LL, Lambert TJ, Frick KM (2007). Age-dependent effects of environmental enrichment on spatial reference memory in male mice. Behav Brain Res.

[83] Magnusson KR, Scruggs B, Aniya J, Wright KC, Ontl T, Xing Y (2003). Age-related deficits in mice performing working memory tasks in a water maze. Behav Neurosci.

[84] Van Praag H, Shubert T, Zhao C, Gage FH (2005). Exercise enhances learning and hippocampal neurogenesis in aged mice. J Neurosci.

[85] Wong AA, Brown RE (2007). Age-related changes in visual acuity, learning and memory in C57BL/6J and DBA/2J mice. Neurobiol Aging.

[86] Klapdor K, Van Der Staay FJ (1997). The Morris water-escape task in mice: strain differences and effects of intra-maze contrast and brightness. Physiol Behav.

[87] Harrison FE, Hosseini AH, McDonald MP (2009). Endogenous anxiety and stress responses in water maze and Barnes maze spatial memory tasks. Behav Brain Res.

[88] Whishaw IQ, Tomie JA (1997). Of mice and mazes: similarities between mice and rats on dry land but not water mazes. Physiol Behav.

[89] Bach ME, Barad M, Son H, Zhuo M, Lu YF, Shih R (1999). Age-related defects in spatial memory are correlated with defects in the late phase of hippocampal long-term potentiation in vitro and are attenuated by drugs that enhance the cAMP signaling pathway. Proc Natl Acad Sci.

[90] Bergado JA, Almaguer W, Rojas Y, Capdevila V, Frey JU (2011). Spatial and emotional memory in aged rats: a behavioral-statistical analysis. Neuroscience.

[91] Doyère V, Gisquet-Verrier P, de Marsanich B, Ammassari-Teule M (2000). Age-related modifications of contextual information processing in rats: role of emotional reactivity, arousal and testing procedure. Behav Brain Res.

[92] Gould TJ, Feiro OR (2005). Age-related deficits in the retention of memories for cued fear conditioning are reversed by galantamine treatment. Behav Brain Res.

[93] Houston FP, Stevenson GD, McNaughton BL, Barnes CA (1999). Effects of age on the generalization and incubation of memory in the F344 rat. Learn Mem.

[94] Kaczorowski CC, Disterhoft JF (2009). Memory deficits are associated with impaired ability to modulate neuronal excitability in middle-aged mice. Learn Mem.

[95] Woodruff-Pak DS, Foy MR, Akopian GG, Lee KH, Zach J, Nguyen KP (2010). Differential effects and rates of normal aging in cerebellum and hippocampus. Proc Natl Acad Sci.

[96] Foster TC, DeFazio RA, Bizon JL (2012). Characterizing cognitive aging of spatial and contextual memory in animal models. Front Aging Neurosci.

[97] Fukushima H, Maeda R, Suzuki R, Suzuki A, Nomoto M, Toyoda H (2008). Upregulation of calcium/calmodulin-dependent protein kinase IV improves memory formation and rescues memory loss with aging. J Neurosci.

[98] Peleg S, Sananbenesi F, Zovoilis A, Burkhardt S, Bahari-Javan S, Agis-Balboa RC (2010). Altered histone acetylation is associated with age-dependent memory impairment in mice. Science.

[99] Mora F, Segovia G, del Arco A (2007). Aging, plasticity and environmental enrichment: structural changes and neurotransmitter dynamics in several areas of the brain. Brain Res Rev.

[100] Rosenzweig ES, Barnes CA (2003). Impact of aging on hippocampal function: plasticity, network dynamics, and cognition. Prog Neurobiol.

[101] Miyakawa T, Yamada M, Duttaroy A, Wess J (2001). Hyperactivity and intact hippocampus-dependent learning in mice lacking the M1 muscarinic acetylcholine receptor. J Neurosci.

[102] Ohira K, Kobayashi K, Toyama K, Nakamura HK, Shoji H, Takao K (2013). Synaptosomal-associated protein 25 mutation induces immaturity of the dentate granule cells of adult mice. Mol Brain.

[103] Hayashi Y, Nabeshima Y, Kobayashi K, Miyakawa T, Tanda K, Takao K (2014). Enhanced stability of hippocampal place representation caused by reduced magnesium block of NMDA receptors in the dentate gyrus. Hippocampus.

[104] Crawley JN, Goodwin FK (1980). Preliminary report of a simple animal behavior for the anxiolytic effects of benzodiazepines. Pharmacol Biochem Behav.

[105] Takao K, Miyakawa T (2006). Light/dark transition test for mice. J Vis Exp.

[106] Komada M, Takao K, Miyakawa T (2008). Elevated plus maze for mice. J Vis Exp.

[107] de Angelis L, File SE (1979). Acute and chronic effects of three benzodiazepines in the social interaction anxiety test in mice. Psychopharmacology (Berl).

[108] File SE (1980). The use of social interaction as a method for detecting anxiolytic activity of chlordiazepoxide-like drugs. J Neurosci Methods.

[109] Porsolt RD, Bertin A, Jalfre M (1977). Behavioral despair in mice: a primary screening test for antidepressants. Arch Int Pharmacodyn Ther.

[110] Ageta-Ishihara N, Yamakado H, Morita T, Hattori S, Takao K, Miyakawa T (2013). Chronic overload of SEPT4, a parkin substrate that aggregates in Parkinson’s disease, causes behavioral alterations but not neurodegeneration in mice. Mol Brain.

[111] Fujioka R, Nii T, Iwaki A, Shibata A, Ito I, Kitaichi K (2014). Comprehensive behavioral study of mGluR3 knockout mice: implication in schizophrenia related endophenotypes. Mol Brain.

[112] Shoji H, Takao K, Hattori S, Miyakawa T (2014). Contextual and cued fear conditioning test using a video analyzing system in mice. J Vis Exp.

[113] Steru L, Chermat R, Thierry B, Simon P (1985). The tail suspension test: a new method for screening antidepressants in mice. Psychopharmacology (Berl).

[114] Onouchi T, Kobayashi K, Sakai K, Shimomura A, Smits R, Sumi-Ichinose C (2014). Targeted deletion of the C-terminus of the mouse adenomatous polyposis coli tumor suppressor results in neurologic phenotypes related to schizophrenia. Mol Brain.

